# High levels of γ-glutamyl hydrolase (GGH) are associated with poor prognosis and unfavorable clinical outcomes in invasive breast cancer

**DOI:** 10.1186/1471-2407-13-47

**Published:** 2013-02-01

**Authors:** Emman Shubbar, Khalil Helou, Anikó Kovács, Szilárd Nemes, Shahin Hajizadeh, Charlotta Enerbäck, Zakaria Einbeigi

**Affiliations:** 1 Sahlgrenska Cancer Center, Department of Clinical Genetics, Institute of Biomedicine, Sahlgrenska Academy at University of Gothenburg, Gothenburg, SE-41345, Sweden; 2 Sahlgrenska Cancer Center, Department of Oncology, Institute of Clinical Sciences, Sahlgrenska Academy at University of Gothenburg, Gothenburg, SE-41345, Sweden; 3 Pathology section, Department of Pathology, Sahlgrenska University Hospital, Gothenburg, SE-41345, Sweden; 4 Department of Clinical and Experimental Medicine, Division of Cell Biology and Dermatology, Linköping University, Linköping, 581 85, Sweden

**Keywords:** GGH, Breast cancer, Primary invasive breast cancer tumors, Prognostic factor, Disease specific survival, Recurrence-free survival

## Abstract

**Background:**

Previously, we performed analysis of gene expression in 46 axillary lymph node negative tumors and identified molecular gene signatures that resulted in different clinical outcomes. The aim of this study was to determine the correlation of γ-glutamyl hydrolase (GGH), fatty acid amide hydrolase (FAAH), Pirin (PIR) and TAF5-like RNA polymerase II, p300/CBP-associated factor (PCAF)-associated factor, 65 kDa (TAF5L), selected from identified gene signatures, with clinical outcomes as well as classical clinicopathological characteristics in primary invasive breast cancer patients.

**Methods:**

The protein levels of GGH, FAAH, PIR and TAF5L were assessed by immunohistochemistry (IHC) on a panel of 80 primary invasive breast tumors. Quantitative real-time PCR (qRT-PCR) and western blot analysis were performed to verify the expression levels of the candidate biomarkers. Patient disease-specific survival (DSS) and recurrence-free survival (RFS) were evaluated using the Kaplan-Meier method. The prognostic biomarkers were identified by univariate analysis with a log-rank test and by multivariate analysis with Cox proportional hazards regression models.

**Results:**

The GGH and FAAH protein levels were significantly up-regulated in invasive breast cancer tumors compared with adjacent non-cancerous tissues. Furthermore, the protein levels of GGH and FAAH were significantly correlated in tumor tissues. Tumoral GGH protein expression was significantly correlated with shorter DSS and RFS. Furthermore, the protein expression of GGH was positively correlated with undifferentiated tumors (BRE grade III) and ER/PR expressing tumors. Multivariate regression analysis showed that only GGH protein expression independently predicts DSS. No such correlations were found for FAAH, PIR and TAF5L protein expression. However, elevated protein levels of FAAH were positively associated with high number of lymph node involvement and upregulated levels of PIR were positively related with lymph node metastasis. The TAF5L was pronouncedly down-regulated in primary invasive breast cancer tissues compared to matched adjacent non-cancerous tissues.

**Conclusion:**

These data show for the first time that cytoplasmic GGH might play a relevant role in the development and progression of invasive breast cancer, warranting further investigations. Our findings suggest that GGH serve as a potential biomarker of unfavorable clinical outcomes over short-term follow-up in breast cancer. The GGH may be a very attractive targeted therapy for selected patients.

## Background

Breast cancer remains a deadly disease and still ranks second among cancer death in women. Clinically, breast cancer represents a heterogeneous and complex disease characterized by uncontrolled cell proliferation and division. The heterogeneity of breast tumors is mirrored in the identification of at least five molecular subtypes including basal-like, luminal A, luminal B, HER2-enriched and normal-like, which are believed to originate from different cell types and follow different progression pathways [1,2]. Several well-established markers including axillary lymph node status, tumour size, histological grade, hormone receptor status, and HER2/*neu* status are used for prognostic evaluation, diagnosis and treatment decisions. However, it should be noted that several novel prognostic markers that fully capture the heterogeneity of breast cancer and do accurately predict it is yet to be identified. Therefore, the identification and clinical implementation of tumour- associated biomarkers for breast cancer to avoid unpleasant side effects with consequent healthcare costs, remains a big challenge.

Previously, we performed gene expression analysis in 46 axillary lymph node negative tumors [3]. Our analysis showed a critical role of 51 genes whose persistently deregulated mRNA levels were significantly associated with unfavorable clinical outcome. The identified 51-gene signature was then evaluated on independent external data sets predicating distance metastasis in breast cancer patients with lymph-node-negative tumours [4]. Of special interest, four genes (*GGH, FAAH, PIR* and *TAF5L*) among the identified 51-gene signature were significantly found to be associated with distant metastases [3,4]. In the present study, the candidate biomarkers *GGH, FAAH, PIR* and *TAF5L* were therefore selected for further analysis. In addition, several publications report their involvement in various cancer forms [5-8].

The GGH catalyzes the hydrolysis of (anti)folylpoly-gamma-glutamates by the removal of gamma-linked polyglutamates [9]. Intracellular folates are essential as cofactors in DNA synthesis and repair, and also required for normal cellular proliferation and replication. Anti- folates are essential as inhibitors of folate-dependent enzymes [10]. They are commonly used in the treatment of various cancer forms, including acute lymphoblastic leukemia, lymphoma, breast cancer, and head and neck cancer [11,12]. Increased expression levels of GGH were reported to be associated with resistance to methotrexate in human sarcoma cell lines [13]. In addition, glutamate was reported to stimulate tumor proliferation and survival via activation of the MAPK and PI3K/Akt pathways in glioma cases [14,15]. The FAAH belongs to a diverse class of enzymes referred to as the amidase signature (AS) family [16]. It regulates the degradation of the main endocannabinoid, anandamide related fatty acid amides [17]. The cannabinoids are bioactive lipids mediators that suggested having a protective effect against tumor growth [18]. The up-regulation of FAAH has been reported to stimulate invasion of prostate carcinoma cells and potentially play a role in prostate tumorigenesis [6]. The PIR is a member of the cupin superfamily [19]. It is a transcriptional regulator that acts as an interactor of nuclear factor I/CCAAT box transcription factor [19]. PIR has also been reported to interact with the proto-oncoprotein Bcl-3, which modulates the activity of NF-κB/Rel transcription factors [20,21]. The TAF5L protein is a component of the PCAF histone acetylase complex which efficiently acetylating histones in a nucleosomal context [22]. The PCAF histone acetylase complex plays a role in the regulation of transcription, cell cycle progression, differentiation and as a tumor suppressor [23].

Taken together, we hypothesized that the candidate biomarkers would allow discriminating tumours according to their aggressiveness. To address this hypothesis, we examined the relationship between *GGH, FAAH, PIR* and *TAF5L* and clinicopathological characteristics, in addition to survival outcomes using a cohort of 80 invasive breast cancer tumors.

## Methods

### Tumor specimens

The current study was done in accordance with the guidelines of the Declaration of Helsinki and approved by the Medical Faculty Research Ethics Committee (Gothenburg, Sweden). Eighty formalin-fixed, paraffin-embedded tissues (FFPE) and fresh-frozen primary invasive breast tissues were obtained from the Departments of Pathology and Oncology at Sahlgrenska University Hospital. The clinicopathological characteristics of the tumors are presented in Table 1. The patients’ follow-up time extended from 1–17 years, and the median survival at the last follow-up was 8 years. Therefore, the primary invasive breast tissues were stratified according to disease-specific survival (DSS) with 8-year censoring. Representative imprints from the fresh-frozen tumors were evaluated for the ratio of cancer/histologically normal cells. The imprints were air dried and stained with May-Günwald-Giemsa (Chemicon, Temecula, CA, USA). The presence of the least 50% cancer cells was required for the specimen to be included in this study.

**Table 1:** Clinicopathological characteristics of 80 invasive breast cancer patients

**Characteristic**	**Long- term survivors ≥8 years**	**Short-term survivors <8 years**
**No. of patients (%)**		
**Mean age (years)**	55 (27–73)	51 (33–72)
**Histologic type**		
Invasive ductal	33 (82.5)	31 (77.5)
Invasive lobular	3 (7.5)	7 (17.5)
Invasive ductal + lobular	0 (0)	1 (2.5)
Other	3 (7.5)	1 (2.5)
Not available	1 (2.5)	0 (0)
**Pathologic tumor size (mm)**		
pT1 (0–20)	8 (20)	8 (20)
pT2 (>20-50)	27 (67.5)	29 (72.5)
pT3 (>50)	5 (12.5)	3 (7.5)
**BRE grade**		
I-II	21 (100)	20 (100)
III	19 (100)	20 (100)
**No. of axillary lymph nodes**		
0	14 (35)	14 (35)
1-3	12 (30)	12 (30)
≥4	14 (35)	14 (35)
**ER/PR receptor**		
Negative	19 (47.5)	20 (50)
Positive	20 (50)	20 (50)
Not available	1 (2.5)	0 (0)
**HER2 status**		
Positive	15 (37.5)	11 (27.5)
Negative	21 (52.5)	20 (50)
Not available	4 (10)	9 (22.5)
**Surgery**		
Lumpectomy	15 (37.5)	15 (37.5)
Mastectomy	25 (62.5)	25 (62.5)
**Radiotherapy**		
No	19 (86)	19 (47.5)
Yes	11 (20)	20 (50)
Not available	2 (9)	1 (2.5)
**Chemotherapy**		
No	12 (36.5)	16 (41)
Yes	17 (51.5)	23 (59)
Not available	4 (12)	0 (0)
**Endocrine therapy**		
No	15 (45.5)	20 (51)
Yes	13 (39.5)	18 (46)
Not available	5 (15)	1 (3)
**Recurrence**		
No	15 (45)	10 (25)
Yes	3 (10)	15 (37.5)
Not available	15 (45)	15 (37.5)

BRE, Bloom, Richardson, Elston/Ellis; HR, hazard ratio; CI, confidence interval; ER/PR, Estrogen/Progesterone receptor. All parameters were coded as 0 (negative) and 1 (positive) except as noted. Histologic type was coded as 1 (ductal), 2 (lobular ductal), 3 (ductal and lobular) and 4 (other).

### Immunohistochemistry (IHC)

Immunohistochemical staining for GGH, FAAH, PIR and TAF5L was carried out in 4-μm tissue sections prepared from formalin-fixed, paraffin-embedded tissue blocks (FFPE).

The FFPE slides were deparaffinised, rehydrated and processed with the Dako EnVision™ FLEX antigen retrieval EDTA buffer pH 9 using DAKO PT Link module (PT Link, Dakocytomation, Denmark) according to the manufacturer’s instructions. The IHC procedure was performed using DAKO stainer (DAKO Auotstainer plus, Dakocytomation, Denmark) following the manufacturer’s instructions. The antibodies employed were rabbit anti-GGH (HPA025226, 1:200; Sigma-Aldrich, Stockholm, Sweden), rabbit anti-FAAH (HPA007425, 1:100; Sigma-Aldrich), rabbit anti-PIR (HPA000697, 1:300; Sigma-Aldrich) and rabbit anti-TAF5L (H00027097-B01, 1:50; Novus Biologicals, Stockholm, Sweden). The scoring of IHC stains in each specimen was evaluated by two pathologists; one of them (AK) reviewed all samples while the other analyzed a portion of the samples. Both pathologists were unaware of the survival outcomes and other clinic-pathological data. The immunoreactivity for GGH, FAAH, PIR and TAF5L was separately analyzed in the malignant epithelial cell compartments of the tumor (cancerous cells) and the adjacent histologically normal epithelial cells (non-cancerous cells) from the same specimens. The immunoreactivity was defined as negative, indicating no staining is observed. The cytoplasmic, membranous or nuclear staining in less than 10% of the cells was also considered as negative. In order to distinguish positive from negative staining as for ER and Her2/*neu* staining, the immunoreactivity was considered as positive, indicating weak, moderate or strong staining in more than 10% of the invasive tumor cells [24,25]. The cytoplasmic, membranous and nuclear staining was classified as either negative or positive for subsequent statistical analyses.

### Fluorescence *in situ* hybridization (FISH)

To assess *HER2/neu* gene status in the 67/80 available fresh-frozen tumor samples, fluorescence *in situ* hybridization was performed. A bacterial artificial chromosome (BAC) clone covering the *HER2/neu* locus (RP11-94 L15) was purchased from BACPAC Resource Center (Oakland, CA, USA, http://bacpac.chori.org/) and used as a FISH probe. FISH was performed on tumor touch-prints prepared from fresh-frozen tumors as described previously [26]. The analysis was performed using a Leica DMRA2 fluorescence microscope (Leica, Stockholm, Sweden) equipped with an ORCA Hamamatsu charged-couple devices camera (Hamamatsu Corporation, Stockholm, Sweden). Scoring of HER2/*neu* hybridization signals was carried out in each tumor specimen by counting the number of signals in at least 100 nuclei. Specimens were scored as either positive (1) when *HER2/neu* gene amplifications were detected in more than 10% of the analyzed cells or negative (0) in all other cases

### Quantitative real-time PCR (qRT-PCR)

QRT-PCR was performed on a cohort of 62/80 tumors which were also used in the IHC analyses. Total RNA was isolated from frozen tumor tissues using TRIzol reagent (Life Technologies, Stockholm, Sweden) and the Qiagen RNeasy mini kit (Qiagen, Stockholm, Sweden) according to the manufacturer’s instructions. DNase treated (Ambion, Texas, US) total RNA was converted subsequently to cDNA using random hexamers and Superscript III (Life Technologies) according to standard procedures. The candidate genes were analyzed using TaqMan Gene expression assays; *GGH* (Hs00914163_m1), *FAAH* (Hs01038660_m1), *PIR* (Hs01125822_m1), *TAF5L* (Hs01039207_m1) from Life Technologies. All assays were carried out in duplicate in a 10 μl reaction volume including: 2 μl of cDNA template, 2x TaqMan Universal PCR Master Mix (ABI, Foster City, USA), and 1x FAM labeled gene-specific assay. All qRT-PCR reactions were performed in 384-well plates using the ABI PRISM 7900HT Sequence Detection System (ABI, Foster City, USA) with an initiation step at 95°C for 10 minutes, followed by 40 cycles at 95°C for 15 seconds and at 60°C for 1 minute. For each assay, a template dilution standard curve (5-fold range) was recorded. The *HPRT1* gene (Hs02800695_m1) was initially selected as an endogenous control based on its constitutive expression using the Illumina HumanHT-12 platform. Moreover, it exhibited low variance in mRNA expression between samples (data not shown). Relative gene expression levels were calculated with the relative standard curve method using CT values of the analyzed genes normalized with *HPRT1*. Genomic DNA and no-template samples were included as controls. Dissociation curves were performed and the samples were run on gels for *GGH*, *FAAH*, *PIR*, *TAF5L* and *HPRT1* to further verify a single band of the correct size.

### Western blotting

Western blotting as previously described [27], was performed on 7 selected cases of invasive breast tumors which were also used in the IHC and qRT-PCR analyses. The rabbit polyclonal antibodies employed were anti-GGH (HPA025226, 1:200; Sigma-Aldrich) and anti-GAPDH (sc-25778, 1:500; Santa Cruz Biotechnology). Imaging analysis was performed using Alpha Ease FC software.

### Statistical analysis

Statistical analyses were performed using SPSS version 20 software. The probabilities of DSS and RFS were estimated by the Kaplan–Meier method, and survival differences were determined by the log-rank test. Survival data were evaluated using univariate and multivariate Cox regression analyses. Cox regression multivariate analysis was performed to identify the independent prognostic factors for predicting DSS. Where appropriate, the chi-square test or t-test was applied to evaluate association. McNemar’s and Exact McNemar’s tests for paired data were used to compare the expression levels of each candidate biomarker in tumor tissues with their matched adjacent non-cancerous tissues.

## Results

### Clinicopathological characteristics

Due to loss of biopsy cores, insufficient tumor cells present in the cores or affluence of necrotic tissue, 72 FFPE specimens out of the collected 80 FFPE specimens were evaluated for GGH, FAAH, PIR and TAF5L immunostaining. The ages of the patients ranged from 27–73 years (median age: 53 years). The tumor sizes ranged from 1.3–7.3 cm (median: 3 cm). Most of the tumors (64/80) were histologically diagnosed as invasive ductal carcinoma, whereas others included invasive lobular carcinoma (10 cases), both type of carcinomas (1 case), other (4 cases), and not available (1 case). For all cases, tumor differentiation was assessed. Of these, 41 tumors were well-to-moderately differentiated (BRE grade I and II), while 39 tumors were poorly differentiated (BRE grade III). The cohort consisted of long-term survivors (46%, ≥8 years) and short-term survivors (54%, <8) following diagnosis. Patients were followed up until March 2011 and during this follow-up period, 1 patient had local recurrence, whereas 17 patients developed distant metastasis (Table 1).

### GGH, FAAH, PIR and TAF5L protein levels in invasive breast cancer and non-cancerous tissues

**Figure 1:**
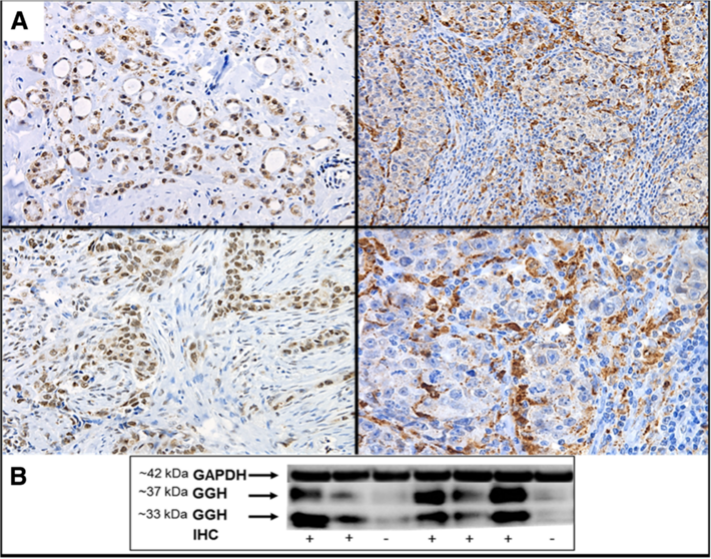
**Detection of GGH protein levels in invasive breast cancer tissues.** (**A**) Immunohistochemical staining of GGH protein expression in primary invasive breast tumors. (**B**) Western blot analysis of GGH protein levels in 7 primary invasive breast cancer tumors. Note: P = GGH-expressing tumor tissues; N = GGH-negative tumor tissues as assessed by immunohistochemistry; IHC = Immunohistochemistry. GAPDH assesses equal loading.

**Figure 2:**
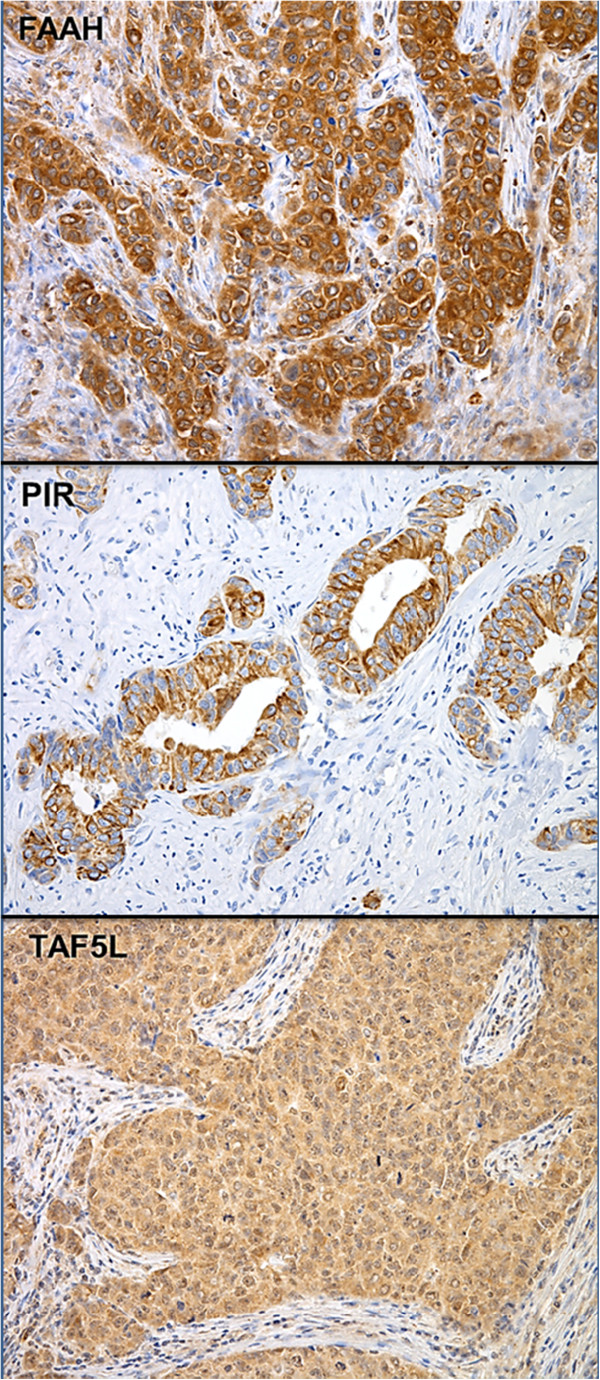
Immunohistochemical detection of FAAH, PIR and TAF5L protein levels in primary invasive breast tumors (A-C).

Breast ductal and lobular epithelial cells exhibited weak to moderate GGH and FAAH staining in the cell cytoplasm. The TAF5L was expressed in the cell nucleus whereas PIR protein was mainly observed in the cytoplasm (92%) or was expressed in both the cytoplasm and cell membrane (8%; Figure 1A and Figure 2). As seen in Table 2, positive expression of GGH was detected in 19% non-cancerous breast tissues, while the remaining tissues had negative staining for GGH. Among the tumor tissues 75% (54/72) of the cases showed positive expression of GGH (χ^2^ = 17.9, *P* < 0.001). Four percent of the FAAH protein expression was positive in non-cancerous breast tissues whereas 89% of the breast cancer tissues expressed FAAH (χ^2^ = 19.3, *P* < 0.001). The frequency and levels of PIR expression was similar between non-cancerous and invasive breast cancer tissues. Eighty-six percent of non-cancerous breast tissues and 85% of the breast cancer tissues were positive for PIR expression. The TAF5L protein levels were elevated in the cell nucleus of the non-cancerous cells compared to the adjacent cancerous cells in the analyzed specimens (χ^2^ = 28.2, *P* < 0.001; Table 2). The expression of GGH was further confirmed by western blot analysis in 7 representative patients whose fresh-frozen tissues were available and a tight correlation between the results of the immunohistochemistry and the western blot analysis were observed. Interestingly, two closely spaced bands corresponding to GGH protein expression were detected at 33- and 37-kD (Figure 1B).

**Table 2:** GGH, FAAH, PIR and TAF5L expression in paired cancer and non-cancer tissues

	**Cancer**	**Non-cancer**	
	**N (%)**	**N (%)**	*P-*value*
**GGH**			
Positive	54 (75)	14 (19)	
Negative	18 (25)	58 (81	<0.001
**FAAH**			
Positive	64 (89)	3 (4)	
Negative	8 (11)	69 (96)	<0.001
**PIR**			
Positive	61 (85)	62 (86)	
Negative	11 (15)	10 (14	1
**TAF5L**			
Positive	32 (44)	67 (93)	
Negative	40 (66)	5 (7)	<0.001

N = number of patients.

* MCNemar test.

### Correlation of GGH, FAAH, PIR and TAF5L protein levels to clinicopathological characteristics of invasive breast cancer

As seen in Table 3, GGH protein expression was significantly associated with high histological tumor grade (BRE grade III, *P* < 0.001), and ER/PR receptors (*P* = 0.01). There was no difference in GGH expression between different pathologic tumor size, histologic tumor type, axillary lymph node status, HER2/*neu* status or age in the cohort’s tissues. A significant association between FAAH expression and patients with more than 4 axillary lymph node metastases (*P* = 0.023) was observed (Table 3). The expression of PIR was positively correlated with lymph node metastasis (*P* = 0.034). The TAF5L protein levels were almost attained statistical significant correlation with the low histological tumor grade (BRE grade I/II; *P* = 0.06), whereas no associations with other clinicopathological parameters were detected (Table 3).

**Table 3:** The association of GGH, FAAH, PIR and TAF5L to the clinicopathological characteristics in addition to clinical outcomes

**Characteristics**	**FAAH**		**PIR**		**TAF5L**		**GGH**	
	**Negative**	**Positive**	**Negative**	**Positive**	**Negative**	**Positive**	**Negative**	**Positive**
	**8 (100)**	**64 (100)**	**11 (100)**	**61 (100)**	**40 (100)**	**32 (100)**	**18 (100)**	**56 (100)**
**Survival ≥8 yrs**	4 (12)	29 (88)	4 (12)	29 (88)	18 (55)	15 (45)	11 (36)	22 (64)
**Survival <8 yrs**	4 (10)	35 (90)	7 (18)	32 (82)	22 (57)	17 (43)	5 (16)	34 (84)
	*P* = 0.55		*P* = 0.53		*P* = 1		*P* = 0.037	
**Recurrence**								
No	3 (12)	22 (88)	2 (8)	23 (92)	16 (64)	9 (36)	9 (36)	16 (64)
Yes	0 (0)	18 (100)	2 (11)	16 (89)	7 (39)	11 (61)	0 (0)	18 (100)
	*P* = 0.29		*P* = 0.87		*P* = 0.12		*P* = 0.009	
**Age**								
27-59	2 (9)	20 (91)	3 (14)	19.(86)	12 (55)	10 (45)	6 (27)	16 (73)
≥60	16 (73)	44 (88)	8 (16)	42 (84)	28 (56)	22 (44)	12 (24)	38 (76)
	*P* = 0.54		*P* = 1		*P* = 1		*P* = 0.78	
**BRE grade**								
I-II	5 (14)	31 (86)	4 (11)	32 (89)	16 (44)	20 (66)	15 (42)	21 (58)
III	3 (8)	33 (92)	7 (19)	29 ( 81)	24 (67)	12 (33)	3 (8)	33 (92)
	*P* = 0.71		*P* = 0.51		*P* = 0.06		*P***<**0.001	
**Axillary lymph node status**								
Negative	2 (8)	22 (92)	7 (29)	17 (71)	14 (58)	10 (42)	5 (21)	19 (79)
Positive	6 (13)	42 (87)	4 (8)	44 (92)	26 (54)	22 (46)	13 (27)	35 (73)
	*P* = 0.71		*P* = 0.034		*P* = 0.81		*P* = 0.88	
**No. of axillary lymph nodes**								
1-3	0 (0)	23 (100)	1 (4)	22 (96)	13 (43)	17 (57)	6 (26)	17 (74)
≥4	6 (24)	19 (76)	3 (12)	22 (88)	10 (56)	8 (44)	7 (28)	18 (72)
	*P* = 0.023		*P* = 0.6		*P* = 0.56		*P* = 0.63	
**ER/PR status**								
Negative	5 (15)	29 (85)	4 (12)	30 (88)	19 (56)	15 (44)	10 (41)	27 (59)
Positive	3 (8)	35 (92)	7 (18)	31.(82)	21 (55)	17 (45)	4 (10)	34 (90)
	*P* = 0.46		*P* = 0.52		*P* = 0.26		*P* = 0.01	
**HER2/*neu*status**								
Negative	5 (14)	32 (86)	7 (19)	30 (81)	20 (54)	17 (46)	10 (71)	4 (29)
Positive	1 (5)	21 (95)	2 (9)	20 (91)	13 (59)	9 (41)	27 (60)	18 (40)
	*P* = 0.4		*P* = 0.46		*P* = 0.79		*P* = 0.53	
**FAAH**								
Negative			2 (18)	9 (82)	4 (50)	4 (50)	5 (28)	13 (72)
Positive			6 (10)	55 (90)	36 (56)	28 (44)	3 (6)	51 (94)
	*-*		*P* = 0.6		*P* = 1		*P* = 0.02	
**PIR**								
Negative	2 (25)	6 (75)			7 (64)	4 (36)	2 (11)	16 (89)
Positive	9 (14)	55 (86)			33 (54)	28 (46)	9 (17)	45 (83)
	*P* = 0.6		*-*		*P* = 0.74		*P* = 0.72	
**TAF5L**								
Negative	4 (10)	36 (90)	3 (27)	8 (73)			5 (28)	13 (72)
Positive	4 (13)	28 (87)	13 (21)	48 (79)			11 (20)	43 (80)
	*P* = 1		*P* = 0.7		-		*P* = 0.53	
**GGH**								
Negative	5 (63)	3 (37)	2 (18)	9 (82)	8 (44)	10 (66)		
Positive	13 (20)	51 (80)	16 (26)	45 (74)	32 (59)	22 (41)		
	*P* = 0.02		*P* = 0.72		*P* = 0.53		*-*	

The association among the levels of GGH, FAAH, PIR and TAF5L proteins was also examined. In tumor tissue samples, seventy-one percent of the tumors had positive expression of GGH and FAAH simultaneously. Seven percent of the tumors had negative expression of GGH and FAAH at the same time. A significant correlation between GGH and FAAH was detected (r = 0.31, *P* = 0.02; Table 3). On the other hand, no association was observed between the expression of PIR and/ or TAF5L and the expressions of the other proteins (Table 3). In non-cancerous tissues, no significant correlation was detected among the expression levels of GGH, FAAH, PIR and TAF5L (data not shown).

### Cytoplasmic expression of GGH is associated with unfavorable clinical outcomes

**Figure 3:**
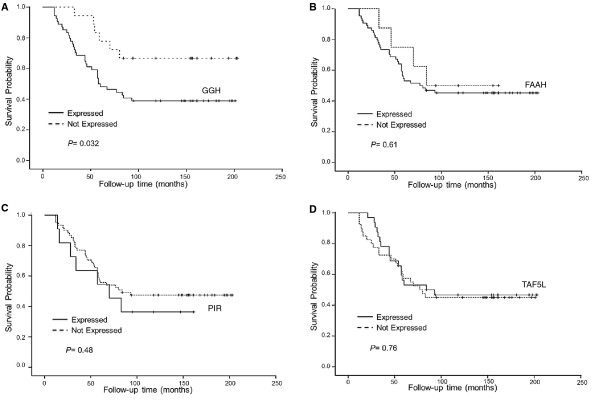
**Kaplan-Meier depicting disease-specific survival in breast cancer according to expression patterns of GGH, FAAH, PIR and TAF5L (A-D).** Dashed line represents patients whose tumors expressed GGH (**A**), FAAH (**B**), PIR (**C**) and TAF5L (**D**). Solid line represents patients whose tumors did not express GGH (**A**-**D**). The *p*-values for the difference between the curves were calculated using log-rank test.

**Figure 4:**
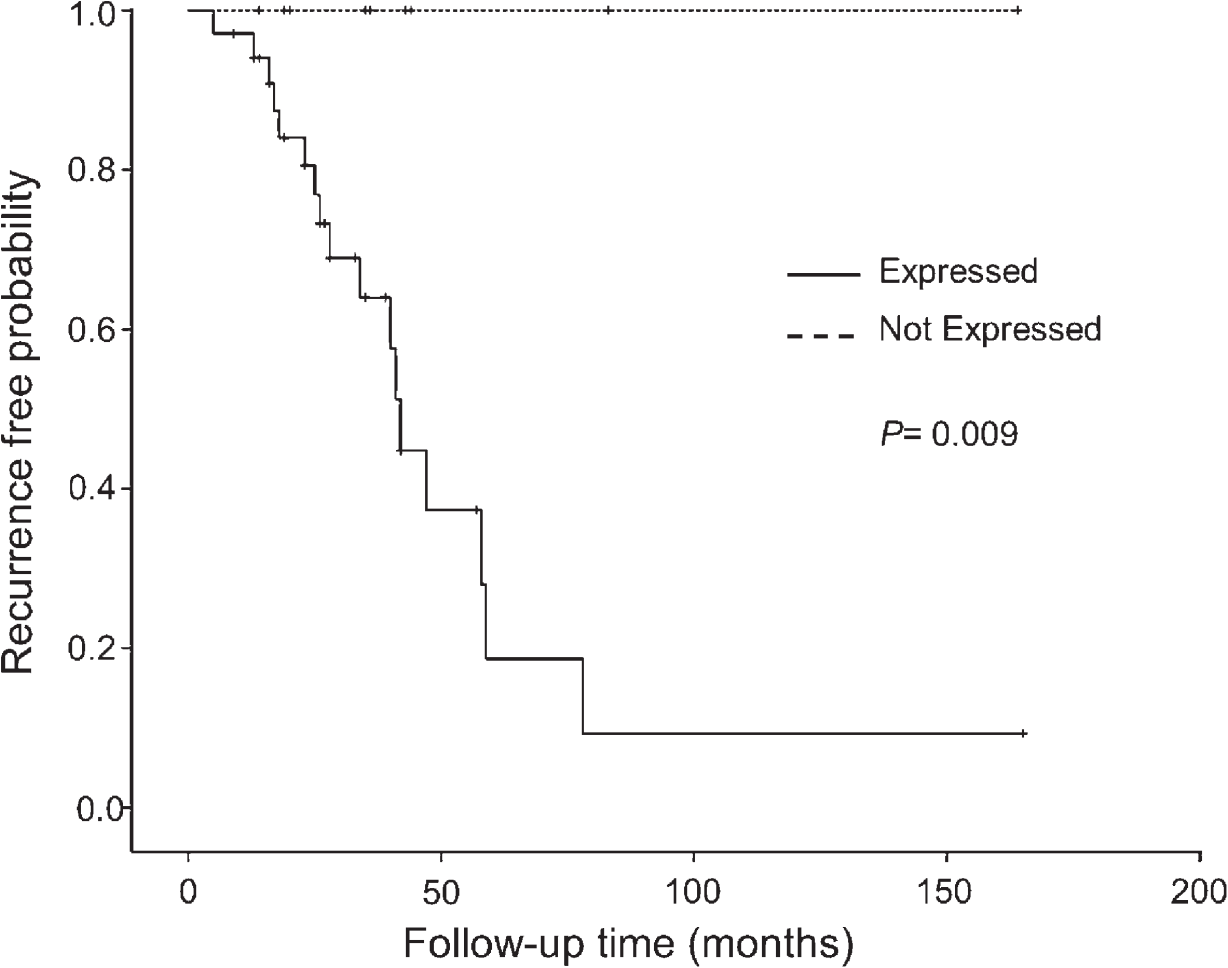
**Kaplan–Meier illustrating the recurrence-free survival of breast cancer patients on the basis of GGH expression levels.** Dashed line represents patients whose tumors expressed GGH and solid line represents patients whose tumors did not. The *p*-values for the difference between the curves were calculated using log-rank test.

The effect of GGH, FAAH, PIR and TAF5L protein expression on patients DSS was conducted by Kaplan-Meier analysis. An inverse correlation between the expression of GGH and 5 year DSS (*P* = 0.024; data not shown) and 8 year DSS (*P* = 0.037; Figure 3) was observed. Eight-year DSS rate was 39% among patients with GGH expressing tumors compared to 68% among patients whose tumors were GGH-negative. Furthermore, the univariate Cox proportional hazards regression analysis revealed that GGH expression exhibited a lower DSS probability with a 2.7 fold higher risk of death (95% CI: 1.0-6.8, *P* = 0.04; Table 4). Moreover, the recurrence-free survival (RFS) of invasive breast cancer patients was also analyzed. Importantly, an adverse association between the expression of GGH and RFS was observed (*P* = 0.009; Table 3). Also, patients with tumors expressing GGH had a 35.5 fold higher risk of recurrence (95% CI: 0.43–2932, *P* = 0.009). As shown in Figure 4, the 8-year RFS rate was 100% in GGH- negative tumors, while it dramatically decreased to 10% in GGH expressing tumors.

**Table 4:** Univariate Cox proportional hazard analysis for disease-specific survival

**Characteristics**	**β**	**SE**	**Wald**	***P*-value**	**HR**	**95.0% CI**	
						**Lower**	**Upper**
GGH	0.98	0.48	4.20	0.04	2.67	1.00	6.80
FAAH	−0.04	0.40	0.02	0.91	0.96	0.44	2.08
PIR	−0.29	0.42	0.49	0.48	0.75	0.33	1.70
TAF5L	−0.10	0.32	0.09	0.76	0.91	0.48	1.71

β: Regression coefficient; SE: standard error of β; HR: hazard ratio; and CI: confidence interval.

Subsequently, all clinicopathological characteristics including age, ER/PR status, HER2/*neu* status, histologic tumor type, pathologic tumor size, histologic tumor grade and axillary lymph node status were enrolled in multivariate survival analysis, which showed that the expression of GGH protein still retained its significance as an independent prognostic factor for unfavorable DSS (95% CI: 1.3–10.3, *P* = 0.02; Table 5). The differences in DSS and RFS based on the protein levels of FAAH, PIR and TAF5L was also examined. As seen in Table 4, no significant association was detected and the expression levels of FAAH, PIR and TAF5L were not significant in univariate analysis.

**Table 5:** Multivariate Cox proportional hazard analysis for disease-specific survival

**Characteristics**	**β**	**SE**	**Wald**	***P*-value**	**HR**	**95.0% CI**	
						**Lower**	**Upper**
AGE	−0.10	0.35	0.03	0.85	0.94	0.47	1.87
GGH	1.20	0.53	5.81	0.02	3.62	1.27	10.3
Histological type	−0.60	0.39	3.09	0.07	0.51	0.24	1.08
BRE grade	−0.05	0.18	0.07	0.78	0.95	0.67	1.34
ER/PR status	0.14	0.35	0.18	0.67	1.12	0.59	2.29
HER2/*neu* status	−0.40	0.38	0.95	0.33	0.69	0.32	1.46
Pathological size	−0.01	0.01	0.23	0.63	0.10	0.97	1.02
Axillary lymph nodes	0.05	0.36	0.02	0.87	1.06	0.52	2.16

β: Regression coefficient; SE: standard error of β; HR: hazard ratio; and CI: confidence interval. BRE: Bloom, Richardson, Elston/ Ellis; ER/PR: Estrogen/Progesterone receptor.

**Figure 5:**
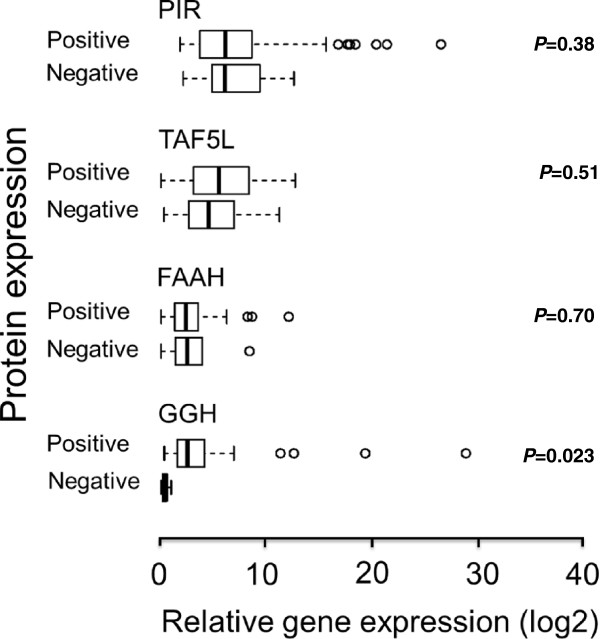
**The relationship between GGH, FAAH, PIR and TAF5L mRNA levels with their corresponding protein levels in breast cancer patients.** The box plots Positive and Negative indicate corresponding gene expression levels for each protein. The mRNA expression of GGH was consistent with the IHC findings. There was no association of FAAH, PIR and TAF5L mRNA levels to their corresponding protein levels.

To further confirm the results of IHC, we assessed GGH, FAAH, PIR and TAF5L mRNA expression levels by qRT-PCR. The GGH mRNA expression was positively correlated with protein levels evaluated by IHC (t-test, *P* = 0.023; Figure 5). The overall concordance between qRT-PCR and IHC data for *GGH* was 92% (57/62). The five discordant tumor samples had high GGH protein levels despite low GGH mRNA levels. In contrast, poor agreement between qRT-PCR and IHC data for *FAAH, PIR* and *TAF5L* were observed (t-test, *P* = 0.70, *P* = 0.51 and *P* = 0.38 respectively; Figure 5).

## Discussion

The identification of novel biomarkers that can help to define individual risk signatures in breast cancer patients are of great clinical value, as they might allow individualized treatments. Previously, we and others have identified candidate molecular gene signatures associated with poor prognosis, including GGH FAAH, PIR and TAF5L [3,4]. In the present study, we investigated the clinical significance of the candidate biomarkers GGH FAAH, PIR and TAF5L in predicting breast cancer progression.

In the present study, we found that the elevated cytoplasmic protein levels of GGH were mainly localized to the tumor cells in comparison to adjacent non-cancerous cells. In urothelial carcinoma of the bladder, elevated level of GGH was also detected in cancerous cells in comparison with non-cancerous cells [5]. In addition, tumoral GGH protein levels were significantly up-regulated in high histological grade tumors in comparison with low histological grade tumors. Taken together, these finding suggest that the expression of GGH is associated with invasiveness and GGH protein levels may increase as the disease progresses. However, detected GGH protein in the non-cancerous tissues may represents the normal function of GGH in maintaining tissue homeostasis of (anti)folate, glutamates or may predict progression of premalignant lesions [28]. High levels of tumoral GGH were also observed to be significantly associated with ER/PR receptors. Eight-year survival of patients with low expression of GGH was significantly better than those with a higher expression. In addition, the multivariate analysis verified that GGH is an independent negative factor in predicting patient DSS as presented by the fact that hazard ratio (HR) for GGH adjusted for age, histological type, histological grade, ER/PR status, HER2/*neu* status, pathologic tumor size, and axillary lymph node status remained significant. These findings suggest that GGH may be involved in promoting carcinogenesis. Furthermore, the elevated levels of GGH was found to be correlated with shorter RFS with more than 35 fold increased risk, suggesting that GGH expression may predict the recurrence behavior of breast cancer. Notably, the association between GGH expression and different cancer forms has been previously reported. The elevated levels of GGH were reported to be correlated with poor clinical outcome in pulmonary neuroendocrine tumors [29]. Elevated plasma level of GGH was observed in patients with metastatic breast cancer in comparison to control subjects and to patients with cancer in remission [30]. High GGH expression level was also detected in hepatoma cells compared with rat hepatocytes [31]. Furthermore, GGH expression was found to act as a prognostic biomarker for acute leukemia in response to methotrexate therapy [32]. Consistent with these findings, our data further suggest that the dysfunction of GGH may play an important role in breast cancer progression and GGH may be an amenable therapeutic target in breast cancer.

In tumor tissues, the mRNA expression of GGH was increased specifically in patients with short-term survivor. This increase corresponded to protein accumulation based on IHC, indicating transcriptional activation. However, the *GGH* gene may be also regulated in tumors at posttranscriptional levels, since the GGH protein in 5 of 62 invasive breast cases was elevated, whereas no increase in mRNA was observed in these samples by qRT-PCR.

The cytoplasmic expression of FAAH was significantly up-regulated in invasive breast tumor tissues compared to the non-cancerous tissues. In addition, the expression levels of FAAH were significantly increased in patients with higher number of axillary lymph node metastases. Up-regulation of FAAH indicates down-regulation of cannabinoids, which play an important role in preventing tumor growth [18]. Taken together, these findings suggest that elevated level of FAAH may promote breast cancer tumors invasion and metastasis. Interestingly, a significant correlation between GGH and FAAH protein expressions was detected in the tumor tissues, whereas no association in the non-cancerous tissues was seen, suggesting that the tumor micro-environmental effects may regulate the expression of GGH and FAAH simultaneously [33]. In addition, GGH accumulation may reflect a functional correlation with FAAH expression, which could play a role in the progression of breast carcinoma. In the current study, expression of PIR in tumor tissues was virtually similar to the non-cancerous tissues. Previous studies reported that some gene expression patterns in the invasive tissues are comparable to their non-invasive breast tissues, suggesting that these signatures may predict progression of early premalignant lesions in non-cancerous tissues [28,34,35]. The PIR functions as a transcriptional regulator whose expression is deregulated in several cancer types. Furthermore, high expression of PIR was reported to be essential to overcome the senescence barrier [36]. We also examined the clinical significance of PIR protein expression. The higher expression of PIR was significantly associated with presence of lymph node metastasis, suggesting that the expression of PIR is associated with invasiveness and supports the reported association of PIR expression with enhanced malignant potential [36,37].

In the present study, the nuclear expression of TAF5L was significantly down-regulated in invasive breast tumor tissues compared to the non-cancerous tissues, which suggests a potential tumor suppressor role in breast cancer. The expression of TAF5L was elevated in patients with low histological grade tumors compared to patients with high histological grade tumors, although the differences were not significant (*P* = 0.06). Furthermore, high mRNA expression levels of TAF5L were detected in 97% of the analyzed tumors, whereas only 56% of the analyzed tumors expressed TAF5L protein. The reduction of TAF5L protein in the analyzed tissues may contribute to disease progression.

The discordant results detected between mRNA and protein expression of FAAH, PIR and TAF5L, could be due to multiple factors including, tumor heterogeneity, posttranscriptional regulation, differences in mRNA and protein turnover rates or poor specificity of the antibody used for IHC [38,39].

In the cohort of 80 patients, even though the expression of FAAH, and PIR did not predict DSS and RFS, the expression of these candidate biomarkers were significantly associated with other clinicopathological characteristics. These results need to be further validated by a larger sample size and additional studies are needed to elucidate the molecular mechanisms through which GGH, FAAH, PIR and TAF5L may participate in the development and progression of breast cancer.

## Conclusion

We demonstrate that elevated protein levels of GGH protein were associated with unfavorable prognosis and poor outcome in an independent cohort of 80 primary invasive breast cancer tumors. Paired breast cancer tissues and adjacent non-cancerous tissues have been found to express GGH differentially, with the cancer tissues displaying significantly higher expression of GGH protein. In addition, our data suggests that GGH is a potential independent prognostic factor of DSS when compared to other widely used prognostic factors. In addition, we have also demonstrated an association between elevated levels of FAAH and PIR and high number of axillary lymph node involvement and lymph node metastasis, respectively. A significant lower expression of TAF5L was observed in tumor cells compared with their adjacent non-cancerous cells. These candidate biomarkers may be useful in improved risk stratification in breast cancer patients. Their prognostic values, however, cannot be reliably assessed from the present analyses and further studies are needed to investigate these possibilities.

## Abbreviations

GGH: γ-glutamyl hydrolase; FAAH: Fatty acid amide hydrolase; PIR: Pirin; TAF5L: TAF5-like RNA polymerase II p300/CBP associated factor (PCAF)-associated factor, 65 kDa; IHC: Immunohistochemistry; CI: Confidence interval; DSS: Disease-specific survival, RFS: recurrence-free survival; FFPE: Formalin-fixed, paraffin-embedded tissues; CI: Concordance index.

## Competing interests

The authors declare that they have no competing interests.

## Authors’ contributions

ES and SH performed the immunohistochemistry. AK evaluated the immunostained breast cancer tissues. AK and ZE provided the clinical information. ES performed the FISH, the western blot and qRT-PCR. ES and SN performed the statistical analysis. ES interpreted the results and wrote the paper. KH, CE and ZE supervised the study. KH, AK, SN, SH, CE and ZE critically revised the manuscript. All authors read and approved the final manuscript.

## Pre-publication history

The pre-publication history for this paper can be accessed here:

http://www.biomedcentral.com/1471-2407/13/47/prepub
